# Phenotypic and Genotypic Identification of Four Cases of Plasmid-Mediated AmpC β-Lactamases-Producing Escherichia coli Admitted to a Tertiary Centre

**DOI:** 10.7759/cureus.64829

**Published:** 2024-07-18

**Authors:** Zeti Norfidiyati Salmuna, Alyaa Farhan Zulkefli, Nik Zuraina Nik Mohd Noor, Nur Saidah Ahmad Bakri

**Affiliations:** 1 Medical Microbiology and Parasitology, Universiti Sains Malaysia School of Medical Sciences, Kota Bharu, MYS

**Keywords:** escherichia coli, ampc induction test, cefoxitin, pampc genotypes, e.coli, pampc β-lactamases

## Abstract

We describe four patients with a positive culture of AmpC β-lactamases-producing* Escherichia coli *(*E. coli*)*, *despite the fact that our understanding of plasmid-mediated AmpC β-lactamases (pAmpC) is currently limited. Three out of four cases of AmpC β-lactamases-producing *Escherichia coli* were isolated from a urine sample, and one was from a peritoneal fluid sample. All four isolates are resistant to cefoxitin disc and were subjected to a confirmatory AmpC phenotypic test (AmpC induction test) and monoplex polymerase chain reaction (PCR) for the determination of six pAmpC genotypes (blaDHA, blaEBC, blaMOX, blaFOX, blaACC, and blaCIT). All four *E. coli* isolates tested negative for the AmpC induction test, while monoplex PCR analysis was positive only for the blaDHA pAmpC genotype and negative for all five other genotypes (blaEBC, blaMOX, blaFOX, blaACC, and blaCIT).

A common clinical characteristic across all patients was fever. One patient was treated for perforated sigmoid diverticulitis, while the other three patients were treated for acute pyelonephritis or urinary tract infections (UTIs). Each patient improved significantly and was successfully discharged.

## Introduction

One of the many harmful and commensal members of the human microbiota is *Escherichia coli* (*E. coli*). It continues to be the most common uropathogen in acute community-acquired uncomplicated urinary tract infections (UTIs) and the principal pathogen responsible for urosepsis, which puts a tremendous amount of strain on healthcare systems around the globe [1]. The problem has been made worse by the fact that, over the past few years, the prevalence of multidrug-resistant *E. coli *has steadily increased. Of particular concern are *E. coli *that produce extended-spectrum β-lactamases (ESBLs) and/or AmpC β-lactamases. This will ultimately result in infections that are more difficult to cure and more expensive.

AmpC β-lactamases, under Ambler class C enzymes, are cephalosporinases that confer resistance against amino-penicillins, cephalosporins, cephamycins (such as cefoxitin and cefotetan), oxyimino-cephalosporins (such as ceftriaxone, cefotaxime, and ceftazidime), and monobactams [2]. AmpC β-lactamases are inhibited by cloxacillin and 3-aminophenylboronic acid; however, unlike ESBLs, AmpC β-lactamase activity is unaffected by ESBL inhibitors like clavulanic acid, and they may be distinguished from ESBLs by their capacity to hydrolyze cephamycins [3].

The production of AmpC β-lactamase is either chromosomal or plasmid-mediated, in which chromosomal AmpC genes are expressed constitutively at a low level [4]. Plasmid-mediated AmpC β-lactamase (pAmpC) is a major challenge to infection control since the AmpC gene is highly transmissible to other bacterial species, can express itself at higher levels [5], and can cause nosocomial outbreaks. 

AmpC β-lactamases producers in the Enterobacterales have been screened using cefoxitin resistance. Nevertheless, changes to the permeability of the outer membrane may potentially act as a mediator of cefoxitin resistance [6]. Thus, to establish AmpC production, confirmatory genotypic and phenotypic testing are required. The AmpC disc induction test has successfully detected the DHA-1 plasmid-mediated gene in *Klebsiella pneumoniae* (*K. pneumoniae*) [7]. The low agreement between the D69C AmpC detection set, cefoxitin-cloxacillin double disk synergy (CC-DDS), and the AmpC induction tests suggests that the lack of an established validated method has hampered the phenotypic detection of AmpC β-lactamases [5]. Apart from that, when an organism has several β-lactamases (such as multiple ESBLs or ESBL-AmpC combos) [8], it can be challenging to identify the β-lactamases phenotypically. Therefore, molecular identification of pAmpC is important. We describe the phenotypic and molecular identifications of four cases of pAmpC-producing *E. coli* that were recruited between January 1 and April 30, 2024, and successfully treated and discharged well from the hospital. The cases were selected as they occurred, without skipping any cases that met the study criteria during that period.

## Case presentation

Case one

A seven-month-old baby girl with no medical history was born term via emergency lower segment cesarean section (EMLSCS) due to poor labor progress. She initially presented with a fever and reduced oral intake for one day, with no other symptoms observed by her mother. A physical examination revealed an infected throat and Grade I tonsils. Urine full examination and microscopic examination (FEME) showed the presence of three to 100 pus cells. Her full blood count (FBC) and C-reactive protein (CRP) results are shown in Table 1. Urine culture and sensitivity (Urine C&S) tests isolated AmpC β-lactamase-producing *E. coli*. She was diagnosed with a UTI, with a differential diagnosis of acute pyelonephritis, and treated with intravenous (IV) cefuroxime (145 mg) three times daily for five days. She was discharged well after six days in the hospital with a prescription for nitrofurantoin syrup (8.5 mg) four times daily for an additional five days. She was seen at a pediatric clinic one month after discharge. She was well and asymptomatic and was advised to come if the symptoms recurred.

**Table 1:** All four patients' full blood count (FBC) and C-reactive protein (CRP) values

Tests	Case 1	Case 2	Case 3	Case 4		Reference range
Full blood count (FBC)						Normal TWCC: 3.4-10.1 X 10^9^/L
Total white cell count (TWCC) on admission	16.2 X 10^9^/L	24.9 X 10^9^/L	14.2 X 10^9^/L	11.9 X 10^9^/L		
TWCC upon discharge	13.7 X 10^9^/L	10.5 X 10^9^/L	11.2 X 10^9^/L	10.6 X 10^9^/L		
Interpretation of TWCC	Reducing trend of TWCC seen in all patients					
C-reactive protein (CRP)						Normal CRP : < 5 mg/L
Admission	122 mg/L	77.3 mg/L	No CRP taken on admission		35.2 mg/L	
Discharge	126 mg/L	No repeat CRP	No CRP taken upon discharge	3.9 mg/L		
Interpretation of CRP	Elevated and static	Elevated	Unable to comment since no CRP was taken	Elevated and reducing in trend		

Case two

A 31-year-old woman with a history of a prolapsed intervertebral disc presented with UTI symptoms, including increased frequency of urination and incomplete voiding, along with left flank pain for three days. Upon examination, she was found to be septic with hypotension, a blood pressure (BP) of 90/56, tachycardic with a heart rate (HR) of 115 beats per minute (bpm), febrile (38 °C), and an oxygen saturation (SpO_2_) of 97% under room air. Abdominal examination revealed tenderness on deep palpation over the left suprapubic and flank areas, but the left renal punch was negative. She was treated for acute left pyelonephritis. An ultrasound of the kidneys, ureters, and bladder (USG KUB) showed no stones. Her FBC and CRP results are shown in Table 1. Urine C&S isolated AmpC β-lactamase-producing *E. coli*. In the ward, she received IV ceftriaxone (2 g) once daily for one week, along with Ural sachets and regular paracetamol. She was discharged well after seven days in the hospital with a prescription for nitrofurantoin (100 mg) four times daily for eight days.

Case three

A 67-year-old woman with a history of hyperlipidemia and hypertension presented with a three-day history of fever and abdominal pain, primarily in the lower abdomen. Other symptoms included post-meal vomiting, anuria for one day, reduced oral intake, and a three-day history of diarrhea. On examination, she was found to be septic with a BP of 126/80, tachycardic (HR: 139 bpm), febrile (38.7 °C), and with a SpO_2_ of 99% under room air. Abdominal examination revealed generalized tenderness with guarding; however, bilateral renal punches were negative. She was diagnosed with perforated sigmoid diverticulitis and underwent a laparotomy Hartmann procedure along with an appendicectomy. Her FBC results are shown in Table 1. Peritoneal fluid culture isolated AmpC β-lactamase-producing *E. coli*. In the ward, she completed IV meropenem (1 g) three times daily and IV metronidazole (500 mg) three times daily for one week. She was discharged well after nine days in the hospital, with advice on stoma care and suture removal in one week and a one-month appointment in the surgical outpatient clinic. Approximately one month following her discharge, she was evaluated at the surgical outpatient clinic. She was in good health and showed no symptoms.

Case four

A 60-year-old woman with an underlying history of diabetes mellitus, hypertension, and a neck of femur fracture presented with fever, intermittent vomiting, and epigastric pain for two days. Upon examination, her vital signs were as follows: BP: 136/68, HR: 102, febrile (37.5°C) with SpO_2_ of 97% under room air. An abdominal examination revealed a positive right renal punch. Her FBC and CRP results are shown in Table 1. Blood and urine C&S isolated AmpC β-lactamase-producing *E. coli*. She was treated for sepsis secondary to right pyelonephritis. A USG KUB showed no stones. In the ward, she received IV cefuroxime for three days, then switched to IV cefepime (1 g) twice daily for one week. She was discharged well after 12 days of hospitalization. She was seen one month later, and she was well and showed no symptoms.

Phenotypic and genotypic results of the four *E. coli* isolates

In all four *E. coli* isolates, 100% (n = 4/4) showed resistance to cefoxitin disc. All isolates were negative for the AmpC induction test. A monoplex polymerase chain reaction (PCR) analysis of cefoxitin-resistant *E. coli* isolates revealed that all isolates (n = 4/4) were positive only for the blaDHA pAmpC genotype and negative for all five other genotypes (blaEBC, blaMOX, blaFOX, blaACC, and blaCIT). A list of primers used for the detection of six pAmpC *E. coli* is obtained from a paper by Perez et al. (2002) [9]. Table 2 summarizes the phenotypic and genotypic results of all four cefoxitin-resistant *E. coli* isolates.

**Table 2:** Phenotypic and genotypic results of four E. coli isolates pAmpC: plasmid-mediated AmpC β-lactamases; PCR: polymerase chain reaction; *E. coli*: *Escherichia coli*

		n (%)	Reference range
Cefoxitin disc screening test (resistant: ≤ 14 mm)*		4/4 (100%)	Sensitive: ≥ 18 mm; intermediate: 15-17 mm; resistant: < 14 mm
Confirmatory phenotypic test (AmpC induction test)		0/4 (0%)	Not applicable
PCR for six pAmpC genes	blaDHA	4/4 (100%)	Not applicable
	blaCIT	0/4 (0%)	
	blaEBC	0/4 (0%)	
	blaMOX	0/4 (0%)	
	blaFOX	0/4 (0%)	
	blaACC	0/4 (0%)	

## Discussion

As AmpC β-lactamases producers in the Enterobacterales have been screened using cefoxitin resistance, the breakpoints used are following Clinical and Laboratory Standards Institute (CLSI) M100 23rd Ed. (2023) [10]. In our study, all four isolates yielded negative results on phenotypic confirmatory tests, highlighting the test's poor specificity and the lack of a validated method for phenotypic AmpC confirmatory testing. The existing phenotypic tests have various advantages and disadvantages, making it challenging to accurately detect AmpC β-lactamase through phenotypic screening carried out in clinical laboratories. Hence, detection by molecular tests is more reliable than phenotypic confirmatory tests.

According to the European Committee on Antimicrobial Susceptibility Testing (EUCAST), PCR is still required to discriminate between plasmid or chromosomal-mediated AmpC [11]. To date, there is no standardized guideline offering phenotypic detection of AmpC β-lactamase. Our findings showed low agreement between phenotypic and genotypic methods, with phenotypic testing underestimating the presence of AmpC enzymes and not correlating well with the molecular results.

One study on the detection of AmpC β-lactamases among Enterobacteriaceae isolates among patients in an Egyptian hospital showed that three phenotypic AmpC confirmatory methods (AmpC E test, disc approximation test, and AmpC EDTA disc test) were able to identify AmpC producers correctly for 38.1% of *E. coli* isolates [4]. The predominant pAmpC genes detected were CMY-1 (under blaMOX), followed by CMY-2 (under blaCIT), and FOX-1 (under blaFOX), respectively, for those tested *E. coli* isolates [4].

A study on genotypic and phenotypic detection of AmpC β-lactamases among Enterobacter species in a Malaysian teaching hospital showed that the positive result was the lowest for the AmpC induction test in comparison to the D69C AmpC phenotypic detection test and CC-DDS, which refers to the cefoxitin-cloxacillin double disk synergy test test for detection of AmpC production in *Enterobacter *spp. [5]. However, we couldn't do both tests (the D69C AmpC phenotypic detection test and the CC-DDS test) for our study as the kit and the disc were not approved by the Medical Device Authority (MDA) for use in Malaysia at the moment. Even though this study focused on Enterobacter isolates, they also found blaDHA apart from blaMIR/ACT for AmpC gene detection, similar to our study that found blaDHA [5].

A study on the evaluation of screening methods to detect pAmpC among *E. coli*, *K. pneumoniae*, and *Proteus mirabilis* showed that the CC-DDS test showed the highest sensitivity and specificity, which was 95% [6]. However, to date, there is no single phenotypic test that can differentiate between AmpC β-lactamases carried by chromosome or plasmid. Among *E. coli* isolates tested for pAmpC genes, this study has predominantly detected blaCIT, followed by blaDHA genes [6]. However, only blaDHA has been found in our patients so far.

Another study regarding the prevalence of AmpC β-lactamases among *E. coli* isolates used the AmpC disc test (EDTA-impregnated filter paper disc) as a phenotypic confirmatory method, followed by pAmpC gene detection using PCR [8]. In this study, they have detected blaCIT as the predominant pAmpC gene, followed by blaMOX and blaEBC, respectively [8]. This study also reported the coexistence of multiple pAmpC genotypes among *E. coli* isolates [8], even though our analysis only identified one pAmpC genotype, blaDHA, among the four isolates tested.

## Conclusions

In conclusion, the only pAmpC β-lactamase gene detected was blaDHA among *E. coli* isolates in our center. The cefoxitin screening test may assist in screening for AmpC β-lactamase enzymes, but the AmpC disc induction test can’t confirm the presence of the enzymes. The presence of the blaDHA gene in all four isolates of *E. coli*, which were tested negative by phenotypic confirmatory tests, indicates low agreement between phenotypic and genotypic methods.
